# Contemporary Endovascular Treatment of Femoropopliteal Disease: Lesion Preparation, Antiproliferative Therapies, and Emerging Technologies

**DOI:** 10.3390/jcm15197651

**Published:** 2026-10-02

**Authors:** María Lourdes del Río Solá, Sandra Pérez Fernández, Sergio Asensio Rodríguez

**Affiliations:** 1 Servicio de Angiología y Cirugía Vascular, Hospital Clínico Universitario de Valladolid, 47003 Valladolid, Spain; 2 Departamento de Cirugía, Facultad de Medicina, Universidad de Valladolid, 47005 Valladolid, Spain; 3 Departamento de Ciencias de la Salud, Universidad Europea Miguel de Cervantes, 47012 Valladolid, Spain

**Keywords:** femoropopliteal disease, peripheral artery disease, drug-coated balloon, sirolimus-coated balloon, drug-eluting stent, atherectomy, intravascular lithotripsy, intravascular ultrasound

## Abstract

**Background**: Endovascular treatment of femoropopliteal atherosclerotic disease has evolved from plain balloon angioplasty and routine stenting toward lesion-oriented strategies that combine plaque assessment, selective vessel preparation, antiproliferative drug delivery, and provisional scaffolding. The increasing availability of drug-coated balloons, drug-eluting stents, atherectomy, intravascular lithotripsy, intravascular imaging, and sirolimus-based technologies has improved therapeutic options but has also made device selection more complex. **Objective**: To synthesize comparative evidence and present a practical lesion-oriented framework for selecting vessel preparation, antiproliferative therapy, and selective scaffolding. **Methods**: A structured narrative review of contemporary evidence was performed, prioritizing randomized controlled trials, head-to-head comparisons, systematic reviews, meta-analyses, and consensus documents relevant to femoropopliteal intervention. **Results**: Lesion morphology—particularly length, chronic total occlusion, calcification pattern, popliteal involvement, and in-stent restenosis—strongly influences procedural strategy and durability. Atherectomy and specialty balloons can optimize acute vessel preparation in selected lesions, although consistent long-term superiority has not been demonstrated. Intravascular lithotripsy has the strongest randomized evidence for heavily calcified lesions, reducing flow-limiting dissection and bailout stenting. Paclitaxel-coated balloons remain a central leave-nothing-behind therapy, with contemporary evidence not confirming the previously proposed late mortality signal. Sirolimus-coated balloons have now entered the field with randomized noninferiority data versus paclitaxel through 2 years. Drug-eluting and other contemporary stents remain important when recoil, dissection, or complex anatomy make a purely scaffold-free strategy unreliable. **Conclusions**: Contemporary femoropopliteal intervention is best approached as a sequence of lesion characterization, selective preparation, antiproliferative therapy, and selective scaffolding. Treatment should be matched to lesion morphology and the final mechanical result, with focal repair or stenting when dissection or recoil compromises flow.

## 1. Introduction

Atherosclerotic disease of the superficial femoral and popliteal arteries remains one of the most technically demanding territories for endovascular therapy. The femoropopliteal segment is exposed to repetitive flexion, torsion, compression, and elongation, while lesion complexity varies widely with respect to length, chronic total occlusion, plaque composition, calcification, vessel diameter, and popliteal involvement. These features influence not only acute luminal gain but also dissection, elastic recoil, drug uptake, stent durability, and late restenosis. Consequently, a single device-based strategy is unlikely to be optimal across the full spectrum of femoropopliteal disease [1,2,3].

The therapeutic landscape has evolved substantially beyond plain balloon angioplasty. Paclitaxel-coated balloons (DCBs) established the principle of delivering antiproliferative therapy without leaving a permanent implant, with randomized trials demonstrating higher primary patency and lower repeat revascularization than conventional angioplasty. Drug-eluting stents (DESs) subsequently provided durable antiproliferative scaffolding, whereas newer approaches have focused on lesion preparation, plaque modification, intravascular imaging, and selective rather than routine stent implantation [4,5,6,7].

This evolution has also changed the clinical question. Rather than asking which device is universally superior, contemporary practice asks which combination of lesion assessment, vessel preparation, drug delivery, and scaffolding is most appropriate for a particular lesion phenotype. This distinction is especially relevant in long lesions, chronic total occlusions, heavily calcified disease, popliteal lesions, and in-stent restenosis, where the mechanical and biological determinants of failure differ substantially [1,2,8,9,10,11,12].

The role of lesion preparation remains a central area of debate. Atherectomy can improve acute luminal gain and reduce severe dissection or bailout stenting in selected lesions, but randomized and pooled data have not consistently demonstrated a durable clinical advantage that would justify routine use in all femoropopliteal interventions. In contrast, intravascular lithotripsy (IVL) has shown a more clearly defined procedural role in moderate-to-severe calcification by modifying deep and superficial calcium while minimizing barotrauma. At the same time, intravascular ultrasound (IVUS) has challenged angiography-only assessment by demonstrating clinically relevant differences in vessel sizing, lesion characterization, and treatment selection [8,9,10,11,12,13,14,15,16,17,18].

Antiproliferative therapy is also entering a new phase. The initial mortality concern surrounding paclitaxel-coated devices has not been confirmed by contemporary randomized evidence and updated meta-analyses, while sirolimus-coated balloon technology now has direct randomized comparison with paclitaxel in femoropopliteal disease. The SIRONA trial and its 2-year follow-up therefore represent an important transition from early single-arm experience with limus-based devices toward comparative clinical evidence [19,20,21,22,23,24,25].

**Figure 1:**
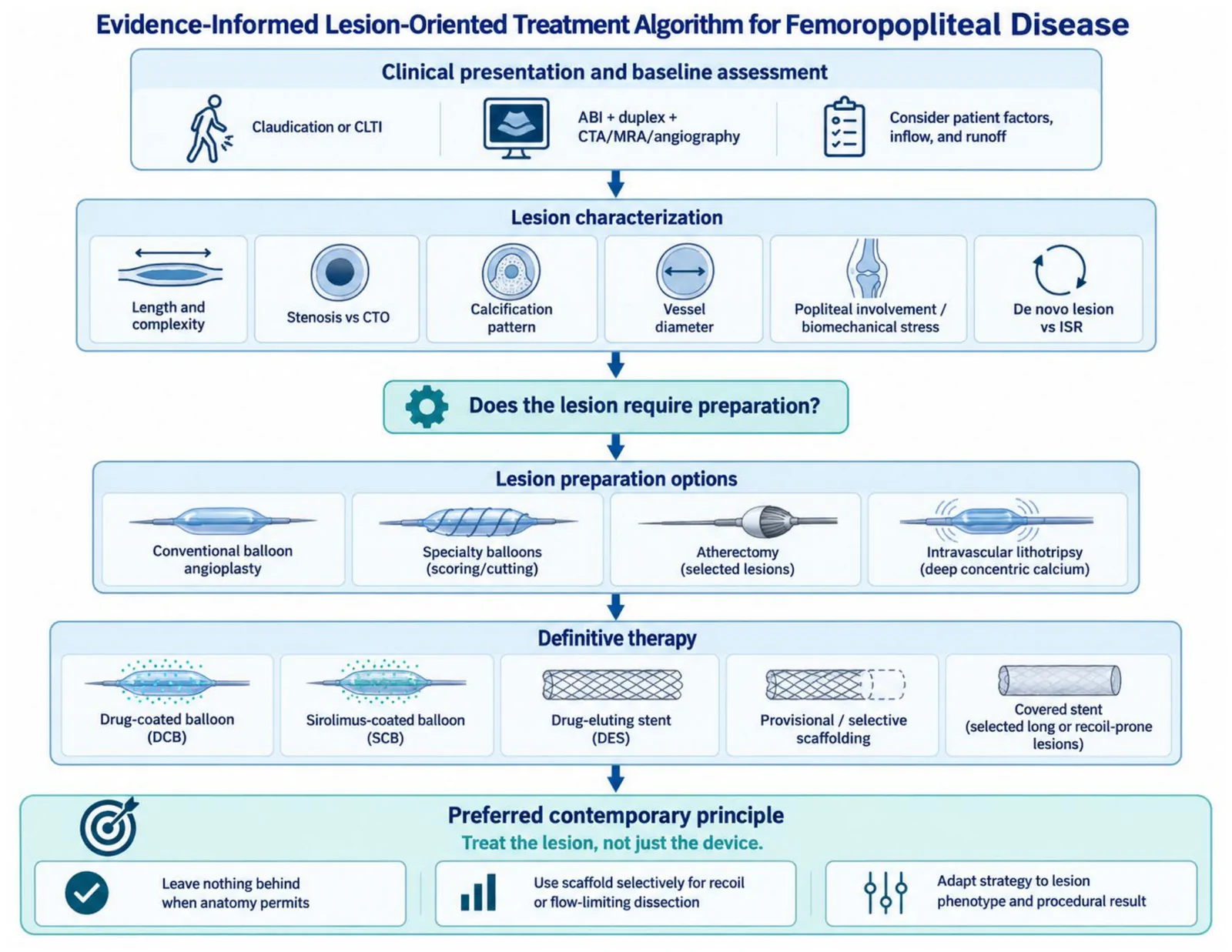
Evidence-informed lesion-oriented treatment algorithm for femoropopliteal disease. The figure summarizes a practical decision pathway that begins with clinical presentation and baseline assessment, proceeds to lesion characterization, and then matches the dominant lesion problem to selective lesion preparation and definitive therapy. The central message is that contemporary treatment should be tailored to lesion phenotype and procedural result rather than to a single preferred device. Abbreviations: ABI—ankle-brachial index; CTO—chronic total occlusion; CLTI—chronic limb-threatening ischemia; CTA—computed tomography angiography; DCB—drug-coated balloon; DES—drug-eluting stent; ISR—in-stent restenosis; MRA—magnetic resonance angiography; SCB—sirolimus-coated balloon.

This state-of-the-art review critically evaluates contemporary endovascular treatment of femoropopliteal atherosclerotic disease, with emphasis on the interaction between lesion morphology, vessel preparation, antiproliferative therapies, and selective scaffolding. Specifically, we synthesize comparative evidence on vessel preparation, drug delivery, and scaffolding; assess how imaging and lesion morphology modify these choices; and propose an evidence-informed pathway for clinical use. The overall lesion-oriented decision pathway proposed in this review is summarized in Figure 1.

## 2. Literature Search Strategy

A structured literature search was designed for a state-of-the-art narrative review. PubMed/MEDLINE, Scopus, and Web of Science were the principal databases used for this review. The contemporary search window was defined as 1 January 2015 to 14 September 2026, supplemented by seminal studies published before 2015 when required to describe pivotal technological development or long-term evidence. Search concepts combined terms for the femoropopliteal territory (“femoropopliteal”, “femoro-popliteal”, “superficial femoral artery”, and “popliteal artery”) with endovascular treatment terms including angioplasty, drug-coated balloon, paclitaxel, sirolimus, drug-eluting stent, atherectomy, intravascular lithotripsy, scoring or cutting balloon, vessel preparation, lesion preparation, intravascular ultrasound, and covered stent.

Evidence was prioritized hierarchically, with randomized controlled trials and direct head-to-head comparisons used for pivotal efficacy and safety claims, followed by systematic reviews and meta-analyses, prospective multicenter studies, and large observational cohorts. Expert consensus documents and contemporary narrative reviews were used primarily to frame treatment pathways and identify areas in which direct comparative evidence remains limited. Studies focused exclusively on aortoiliac, common femoral, infrapopliteal, aneurysmal, acute thromboembolic, or non-atherosclerotic disease were not considered central to the review.

## 3. Why Femoropopliteal Lesion Morphology Matters

The femoropopliteal segment is not a uniform therapeutic territory. Treatment durability is influenced by the interaction between plaque biology, lesion geometry, vessel diameter, lesion length, occlusion, calcification, and the unique mechanical environment of the superficial femoral and popliteal arteries. These variables affect not only late restenosis but also the immediate procedural result: crossing success, balloon expansion, residual stenosis, recoil, dissection, drug transfer, and the need for scaffolding. Contemporary expert frameworks therefore place lesion characterization before vessel preparation and definitive therapy, reflecting a transition from device-first treatment toward lesion-oriented strategy selection [1,8].

This distinction is clinically relevant because technologies that perform well in short, mildly calcified stenoses may be less effective in long occlusions, severely calcified plaques, small-caliber vessels, or lesions extending into the popliteal artery. Conversely, aggressive plaque modification may add little to a short, compliant lesion that can be adequately prepared with conventional balloon angioplasty. A contemporary strategy should therefore identify the dominant mechanism that could compromise the result—insufficient luminal gain, recoil, uncontrolled dissection, impaired drug uptake, or inadequate scaffold expansion—and select lesion preparation accordingly [1,2,3,8,9].

### 3.1. Lesion Length and Complexity

Lesion length remains one of the most reproducible markers of procedural complexity and long-term loss of patency. Longer lesions are more frequently associated with chronic total occlusion, diffuse calcium, smaller distal reference diameter, multilevel disease, and a greater probability of subintimal crossing. They also expose a larger arterial surface to barotrauma and drug delivery and, when scaffolding is required, increase the length of permanent implant exposed to flexion and torsion. These factors explain why outcomes reported in pivotal trials of relatively short lesions cannot be extrapolated directly to contemporary real-world disease [26,27].

The available pooled evidence demonstrates a clear durability gradient with increasing lesion complexity. In the contemporary meta-analysis by Sayfo et al., primary patency in long femoropopliteal lesions declined from approximately 85% at 6 months to 42% at 36 months, while freedom from target lesion revascularization fell from approximately 93% to 63% over the same period [27]. The earlier systematic review by Dubosq-Lebaz et al. similarly showed that increasing lesion length is associated with less durable endovascular outcomes [26]. These findings are particularly important when interpreting device trials: a favorable 12-month result in a short lesion should not automatically be used to justify the same strategy in diffuse disease.

From a procedural perspective, lesion length should therefore influence both preparation and definitive therapy. A short, non-calcified stenosis may require little more than controlled balloon preparation before antiproliferative treatment, whereas a long lesion may require staged dilatation, selective plaque modification, careful reassessment of recoil and dissection, and a lower threshold for focal or extended scaffolding when the angiographic result is unstable. Network meta-analyses suggest that several contemporary technologies outperform plain balloon angioplasty, but rankings vary with follow-up duration and lesion complexity, underscoring that no single device has demonstrated universal superiority across all femoropopliteal phenotypes [28,29,30].

### 3.2. Chronic Total Occlusion

Chronic total occlusion (CTO) represents more than an extreme degree of stenosis. CTOs introduce additional technical variables related to crossing strategy, re-entry, guidewire position, vessel sizing, and the extent of vessel-wall injury. Intraluminal crossing may preserve native arterial architecture, whereas subintimal recanalization can create long dissection planes and uncertainty regarding true vessel dimensions. CTOs also commonly coexist with long lesion length and heavy calcification, so the adverse effect attributed to occlusion is often inseparable from overall lesion complexity [1,2,3,26,27].

The procedural consequence of CTO morphology is therefore more important than the binary label itself. After crossing, the operator must determine whether the reconstructed channel is adequately expanded, whether the distal and proximal reference segments have been correctly sized, and whether there is clinically relevant recoil or flow-limiting dissection. A technically successful crossing does not guarantee an adequate platform for drug delivery. In lesions with extensive subintimal passage, residual recoil, or long spiral dissection, a purely leave-nothing-behind strategy may be less predictable and selective scaffolding may be required. Conversely, successful intraluminal crossing followed by adequate vessel preparation may permit treatment without a permanent implant [1,8,28,29,30].

CTO should therefore be viewed as a modifier of treatment strategy rather than an automatic indication for a specific device. The central question after recanalization is whether the lesion has been transformed into a vessel segment that can accept definitive antiproliferative therapy with an acceptable acute mechanical result.

### 3.3. Calcification

Calcification is one of the most important determinants of acute failure in femoropopliteal intervention. Severe calcium can limit balloon expansion, increase residual stenosis, promote uncontrolled dissection, impair stent expansion, and reduce effective contact between a drug-coated balloon and the arterial wall. It may also lead operators to use higher balloon pressures, potentially increasing barotrauma without adequately modifying deep or circumferential calcium [8,9,13,14,15]. Angiographic calcium severity can be recorded with the Peripheral Arterial Calcium Scoring System (PACSS) or the Peripheral Academic Research Consortium (PARC) classification, using standardized categories rather than an unqualified label of “severe calcium” [31,32]. When IVUS is available, the calcium arc and longitudinal extent should be documented to distinguish eccentric from circumferential disease; IVUS cannot reliably measure calcium thickness because acoustic shadowing obscures the deeper border [31]. These descriptors inform preparation, but no single grading threshold has been prospectively validated as a mandatory device-selection rule.

Importantly, calcium should not be treated as a binary variable. Eccentric superficial calcium, concentric or circumferential calcium, long calcified segments, and nodular or bulky plaque create different mechanical problems. A focal eccentric plaque may be amenable to directional debulking, whereas deep circumferential calcium may be better suited to a technology designed to fracture calcium without relying on high-pressure luminal expansion. This concept underpins plaque morphology-based treatment algorithms and explains why the same preparation strategy is unlikely to be optimal for every calcified lesion [8,13].

The clinical relevance of calcium extends beyond acute technical success. Inadequate modification may compromise subsequent antiproliferative therapy by limiting luminal gain or reducing drug–wall contact. Similarly, placing a stent in a severely calcified segment without adequate preparation risks underexpansion and persistent mechanical stress. For this reason, calcification is the clearest example of why lesion preparation and definitive therapy should be considered as two separate—but sequential—decisions. The first decision is how to obtain a compliant, adequately expanded vessel; the second is how to preserve that result [8,9,10,13,14,15].

### 3.4. Popliteal Involvement and Biomechanical Stress

The femoropopliteal artery is exposed to forces that are unusual in most other vascular territories. During hip and knee movement, the vessel undergoes shortening, elongation, torsion, bending, and compression. These forces become particularly relevant in the distal superficial femoral artery and popliteal artery, where a permanent implant must tolerate repeated deformation throughout the gait cycle. Although contemporary nitinol and interwoven stent platforms have improved flexibility and fracture resistance, the biomechanical environment remains a major consideration when deciding whether to implant a scaffold [33,34].

This is one reason why leave-nothing-behind strategies are attractive in popliteal disease: when adequate luminal gain can be achieved without persistent recoil or flow-limiting dissection, avoiding a permanent implant may reduce exposure to long-term deformation-related complications. However, the opposite extreme is equally problematic. Persistently underexpanded or dissected vessels treated without scaffolding may thrombose or restenose despite successful drug delivery. Thus, the decision should not be framed as stent avoidance versus stenting, but as whether the acute mechanical result is sufficiently stable to permit a non-scaffold strategy [1,2,3,33]. For a localized flow-limiting dissection after DCB, a short nitinol tack can provide focal repair while limiting the length of permanent metal; TOBA III evaluated this approach after DCB in the superficial femoral and proximal popliteal arteries [35]. Its single-arm design does not establish superiority over conventional bailout bare-metal or drug-eluting stents. Longer dissections, persistent recoil, or an inadequate lumen may still require a conventional scaffold.

For this reason, ‘leave nothing behind’ should be regarded as a desirable treatment objective rather than a procedural dogma. In selected lesions, especially those involving highly mobile arterial segments, avoiding a scaffold is rational; in others, focal dissection repair or an appropriately selected stent may provide a more stable acute result; comparative long-term durability remains uncertain.

### 3.5. In-Stent Restenosis

Femoropopliteal in-stent restenosis (ISR) should be regarded as a distinct disease substrate rather than simply another stenotic lesion. The mechanism may involve diffuse neointimal hyperplasia, focal edge restenosis, chronic stent underexpansion, stent fracture, neoatherosclerosis, or complete occlusion. These mechanisms have different implications for treatment, and the original stent also constrains subsequent vessel expansion and limits the ability to modify deep plaque directly [36,37].

Plain balloon angioplasty alone is generally associated with high recurrence in femoropopliteal ISR. Contemporary comparative evidence supports DCB and covered-stent strategies over plain balloon angioplasty, while laser or other debulking approaches may add benefit in selected complex or occlusive ISR [36]. The meta-analysis by Cheng et al. further supports interest in debulking before DCB, but the available studies remain heterogeneous and do not justify routine debulking for every restenotic stent [37].

A morphology-based approach is therefore particularly important in ISR. Focal hyperplasia in an otherwise well-expanded stent is a different problem from diffuse restenosis associated with fracture or underexpansion. When feasible, identifying the mechanism of failure should precede selection of another drug-coated or scaffold-based therapy.

### 3.6. Role of Intravascular Imaging

Angiography remains the default imaging modality for femoropopliteal intervention, but it provides a two-dimensional luminogram rather than direct characterization of the vessel wall. Reference-vessel diameter, plaque burden, calcium distribution, true lesion length, and residual disease may therefore be underestimated. These limitations become clinically relevant when device sizing, assessment of stent expansion, or the distinction between recoil and residual plaque determines the next procedural step [16,17,18].

The randomized trial by Allan et al. provides the strongest direct evidence in this territory. In 150 femoropopliteal interventions, IVUS altered treatment in approximately 79% of cases and resulted in larger measured vessel diameters than angiography alone. At 12 months, freedom from binary restenosis was 72.4% with IVUS guidance compared with 55.4% with angiographic guidance (*p* = 0.008), although clinically driven target lesion revascularization was not significantly different [16]. These findings suggest that the principal value of IVUS is not simply diagnostic; it changes sizing and treatment decisions in ways that may translate into improved vessel-level outcomes.

Subsequent systematic reviews and meta-analyses have reinforced the potential value of IVUS-guided femoropopliteal intervention, particularly for lesion characterization, device sizing, and assessment of the final result [17,18]. Optical coherence tomography can provide high-resolution information on luminal and superficial plaque morphology, but its evidence base in the femoropopliteal segment is much smaller and its practical use is limited by field-of-view and blood-clearance requirements [17]. Before intervention, duplex ultrasound defines hemodynamic significance, runoff, and surveillance baselines; CTA or MRA can map lesion length, calcification, access, and multilevel disease when a wider anatomical overview is needed. During treatment, digital subtraction angiography assesses crossing and flow, whereas IVUS adds cross-sectional vessel sizing, calcium arc, wire position, residual stenosis, and stent expansion [16,17,18]. Imaging should be selected for the clinical question and should not delay urgent limb revascularization.

Intravascular imaging should therefore be viewed as a strategy-enabling technology rather than a stand-alone treatment. Its greatest potential value is in complex lesions where angiography alone leaves uncertainty regarding vessel size, calcium distribution, stent expansion, or the mechanism of an unsatisfactory result.

## 4. Contemporary Lesion Preparation

Lesion preparation is the mechanical phase that precedes definitive antiproliferative therapy or scaffolding. Its purpose is not merely to enlarge the lumen but to create a predictable substrate for the next treatment step: adequate luminal gain, controlled plaque fracture, minimized recoil, limited dissection, and sufficient vessel-wall contact for drug transfer. The contemporary concept is therefore selective rather than routine. Preparation should address the dominant mechanical problem posed by the lesion, and the intensity of preparation should be proportional to lesion complexity [1,8,9,10,11,12].

This distinction is important because technologies used for vessel preparation differ substantially in mechanism. Conventional balloons redistribute plaque and stretch the arterial wall; specialty balloons concentrate force; atherectomy removes or modifies plaque; laser combines photoablative and mechanical effects; and IVL fractures calcium using acoustic pressure waves. These mechanisms are not interchangeable. The choice should be driven by whether the principal problem is resistant fibrotic plaque, bulky eccentric disease, concentric calcium, long occlusion, or failure of conventional dilatation [8,9,10,13,14,15]. The practical characteristics, preferred lesion phenotypes, advantages, and limitations of the principal preparation technologies are summarized in Table 1.

### 4.1. Conventional Balloon Angioplasty

Plain balloon angioplasty remains the fundamental mechanical maneuver in femoropopliteal intervention and continues to serve as the comparator for most newer technologies. Its advantages are simplicity, availability, low profile, and the ability to provide immediate information about lesion compliance. However, conventional angioplasty produces luminal gain through a combination of plaque compression, plaque fracture, and vessel stretch, and these effects are not always controlled. Elastic recoil, residual stenosis, and dissection therefore remain common limitations, particularly in long, fibrotic, or calcified lesions [4,5,6,38].

**Table 1:** Practical lesion-preparation technologies for femoropopliteal disease. Evidence citations and evidence limitations are shown in the Practical Use column.

Technology	Best-Suited Lesion Phenotype	What It Adds	Main Limitations/Risks	Practical Use
**Conventional balloon angioplasty**	Short, non-calcified or compliant lesions; baseline preparation	Simple lumen gain and assessment of vessel response	Recoil; uncontrolled dissection; poor durability if used alone	Default first step. If the result is satisfactory, proceed to drug therapy; scaffold only for mechanical failure. Evidence: DCB trials [4,5,6].
**Scoring/cutting/focal-force balloon**	Fibrotic, balloon-resistant or focal non-circumferential lesions	Controlled plaque modification; less balloon slippage	Limited comparative evidence; added cost	Use for persistent waist/underexpansion before escalating to debulking or IVL. Evidence: morphology consensus [1,8]; device-specific comparative data remain limited.
**Directional atherectomy**	Eccentric bulky plaque; selected nodular or superficial calcium	Debulking and acute luminal gain; may reduce severe dissection/bailout stenting	Distal embolization; cost; no consistent universal long-term benefit	Selective when bulky eccentric plaque prevents adequate lumen or drug delivery [10,11,12,39].
**Rotational/orbital atherectomy**	Diffuse calcified or markedly resistant plaque	Ablation that can facilitate subsequent balloon expansion	Embolic/perforation risk; platform heterogeneity; limited head-to-head RCTs	Selective option when resistant calcified plaque remains poorly compliant. Evidence: synthesis [9,10,11,12]; platform-specific data remain heterogeneous.
**Laser atherectomy**	ISR, occlusive restenosis and selected CTO	Photoablation of neointimal/occlusive tissue	Specialized equipment; embolization/perforation; limited de novo evidence	Primarily an ISR/selected CTO tool rather than routine de novo preparation [36,37]. Evidence: ISR meta-analyses [36,37].
**Intravascular lithotripsy**	Moderate-to-severe concentric or deep calcification	Low-pressure fracture of superficial/deep calcium; fewer severe dissections and less bailout stenting	Cost; long-term comparative superiority and cost-effectiveness uncertain	Preferred calcium-modification option when deep/circumferential calcium limits compliance [13,14,15].

The modern role of plain balloon angioplasty is consequently different from its historical role. In many lesions it is better considered a preparation step than a definitive therapy. A satisfactory response to appropriately sized balloon dilatation may identify a lesion suitable for DCB treatment without additional modification. Conversely, persistent waist, significant recoil, or flow-limiting dissection signals that the lesion has not been adequately prepared and that escalation—specialty balloon, atherectomy, IVL, or scaffolding—may be required.

Balloon sizing and inflation technique also matter. Oversizing and aggressive high-pressure inflation may achieve short-term luminal gain at the cost of deeper arterial injury, whereas undersizing may leave residual stenosis and poor drug contact. Although precise protocols differ across studies and devices, the conceptual goal is controlled expansion rather than maximal barotrauma.

### 4.2. Specialty Balloons

Scoring, cutting, and focal-force balloons are designed to modify plaque in a more controlled fashion than conventional balloons by concentrating dilatation force along defined contact points. This may be particularly useful in resistant fibrotic lesions, focal calcification, or lesions that demonstrate a persistent balloon waist despite conventional dilatation. By focusing force, these devices aim to improve luminal gain while reducing the need for uniformly high balloon pressure [1,8].

Their theoretical appeal is clear, but the clinical evidence is less mature than for DCB, DES, or IVL. Available studies are heterogeneous with respect to lesion phenotype, balloon design, adjunctive therapies, and endpoints, and there is no robust evidence supporting routine use in all femoropopliteal lesions. Contemporary algorithms therefore position specialty balloons as selective lesion-preparation tools rather than definitive therapies [1,8,9].

A practical role is in lesions where conventional angioplasty has demonstrated inadequate compliance, but the morphology does not clearly require debulking or dedicated calcium-fracture therapy. In this setting, a focal-force strategy may offer an intermediate escalation step before more complex plaque-modification technologies.

### 4.3. Atherectomy

Atherectomy includes directional, rotational/orbital, and laser-based technologies, but the term encompasses devices with different mechanisms, procedural goals, and complication profiles. Directional atherectomy physically excises plaque, rotational/orbital systems ablate or sand the luminal surface, and excimer laser atherectomy combines photoablative and mechanical effects. The common rationale is to reduce plaque burden or alter lesion compliance before balloon-based drug delivery or scaffolding [8,9,10,11,12].

The strongest and most consistent evidence for atherectomy concerns acute procedural optimization rather than unequivocal improvement in long-term clinical outcomes. Meta-analyses of randomized trials show reductions in bailout stenting and flow-limiting dissection with atherectomy-based vessel preparation [10,11]. The meta-analysis by Cao et al., comparing atherectomy plus DCB with DCB alone, similarly found lower rates of bailout stenting and dissection but no consistent advantage in 12-month patency or target lesion revascularization, while distal embolization remained an important trade-off [12].

The DEFINITIVE AR randomized pilot trial illustrates this distinction. Directional atherectomy followed by DCB improved the acute angiographic result and reduced severe dissection and the need for bailout stenting compared with DCB alone, but the study was not powered to establish superiority for durable clinical endpoints [39]. Thus, the trial supports atherectomy as a preparation strategy in selected complex lesions, not as evidence for routine debulking before every DCB.

Patient and lesion selection are therefore essential. Atherectomy is most conceptually attractive when plaque burden itself is the dominant mechanical obstacle—for example, bulky eccentric disease, severely fibrotic lesions, selected calcified plaques, or restenotic tissue that limits expansion. Its disadvantages include procedural complexity, cost, device-specific learning curves, risk of perforation or distal embolization, and the potential for overtreatment of lesions that would respond adequately to balloon preparation alone [8,9,10,11,12,39].

Laser atherectomy deserves particular distinction because its evidence is concentrated in selected CTO and ISR scenarios rather than routine de novo disease. In ISR, debulking may improve the luminal substrate before DCB, especially in occlusive or diffuse restenosis, but current pooled evidence remains insufficient to recommend universal debulking [36,37].

### 4.4. Intravascular Lithotripsy

IVL provides a fundamentally different approach to calcified plaque. Rather than removing plaque or relying on high-pressure balloon expansion, the device generates acoustic pressure waves that fracture superficial and deep calcium at relatively low balloon pressure. This mechanism is particularly attractive in concentric or deep calcification, where conventional angioplasty may fail to achieve adequate expansion and atherectomy may preferentially modify only the luminal surface [13,14,15].

The randomized Disrupt PAD III trial provides the clearest comparative evidence. Among 306 patients with moderate-to-severe calcified femoropopliteal lesions, procedural success was higher with IVL than with conventional PTA (65.8% vs. 50.4%; *p* = 0.01). Flow-limiting dissection occurred less frequently (1.4% vs. 6.8%; *p* = 0.03), and stent implantation was substantially reduced (4.6% vs. 18.3%; *p* < 0.001) [15]. These results are clinically meaningful because they demonstrate that calcium modification can improve the quality of the acute result without requiring more aggressive balloon pressure or routine scaffolding.

Subsequent systematic evidence has reported high procedural success and low rates of severe dissection, perforation, and bailout stenting with IVL [14]. Meta-analytic comparisons in moderate-to-severe calcification also support lesion-modification strategies, although heterogeneity in lesion definitions and comparator technologies limits definitive ranking among IVL, atherectomy, and other preparation approaches [13].

IVL should therefore be viewed as a lesion-preparation technology rather than a definitive antiproliferative treatment. Its most evidence-supported role is in lesions where deep or circumferential calcium is expected to compromise balloon expansion, drug transfer, or subsequent stent deployment. Important unresolved issues include cost-effectiveness, direct head-to-head comparison with specific atherectomy platforms, and the durability of different IVL-based combination strategies [13,14,15].

### 4.5. Combination Strategies: Preparation Before Drug Delivery

The rationale for combined therapy is straightforward: if restenosis prevention depends on adequate drug transfer, then the vessel must first be expanded sufficiently and severe dissection or recoil must be controlled. Plaque modification may increase acute luminal gain, improve balloon apposition, and reduce the need for unplanned scaffolding. However, mechanistic plausibility should not be confused with proven clinical superiority.

The network meta-analysis by Yiu et al. and pairwise analyses of atherectomy-based strategies consistently suggest that more intensive vessel preparation can improve procedural endpoints, particularly bailout stenting and flow-limiting dissection [9,10,11,12]. In contrast, durable advantages in primary patency or target lesion revascularization are less consistent. This discrepancy is important: a cleaner angiographic result is valuable, but it does not automatically justify the additional procedural complexity or cost of routine plaque modification.

Combination therapy should therefore be phenotype-driven. In a compliant, non-calcified lesion, conventional balloon preparation followed by antiproliferative therapy may be entirely adequate. In bulky eccentric plaque, atherectomy may facilitate expansion. In deep circumferential calcium, IVL may provide a more mechanistically appropriate preparation strategy. In resistant fibrotic disease, a specialty balloon may be sufficient. The goal is not to maximize the number of devices used but to solve the specific mechanical problem that would otherwise compromise definitive treatment [1,8,9].

**Figure 2:**
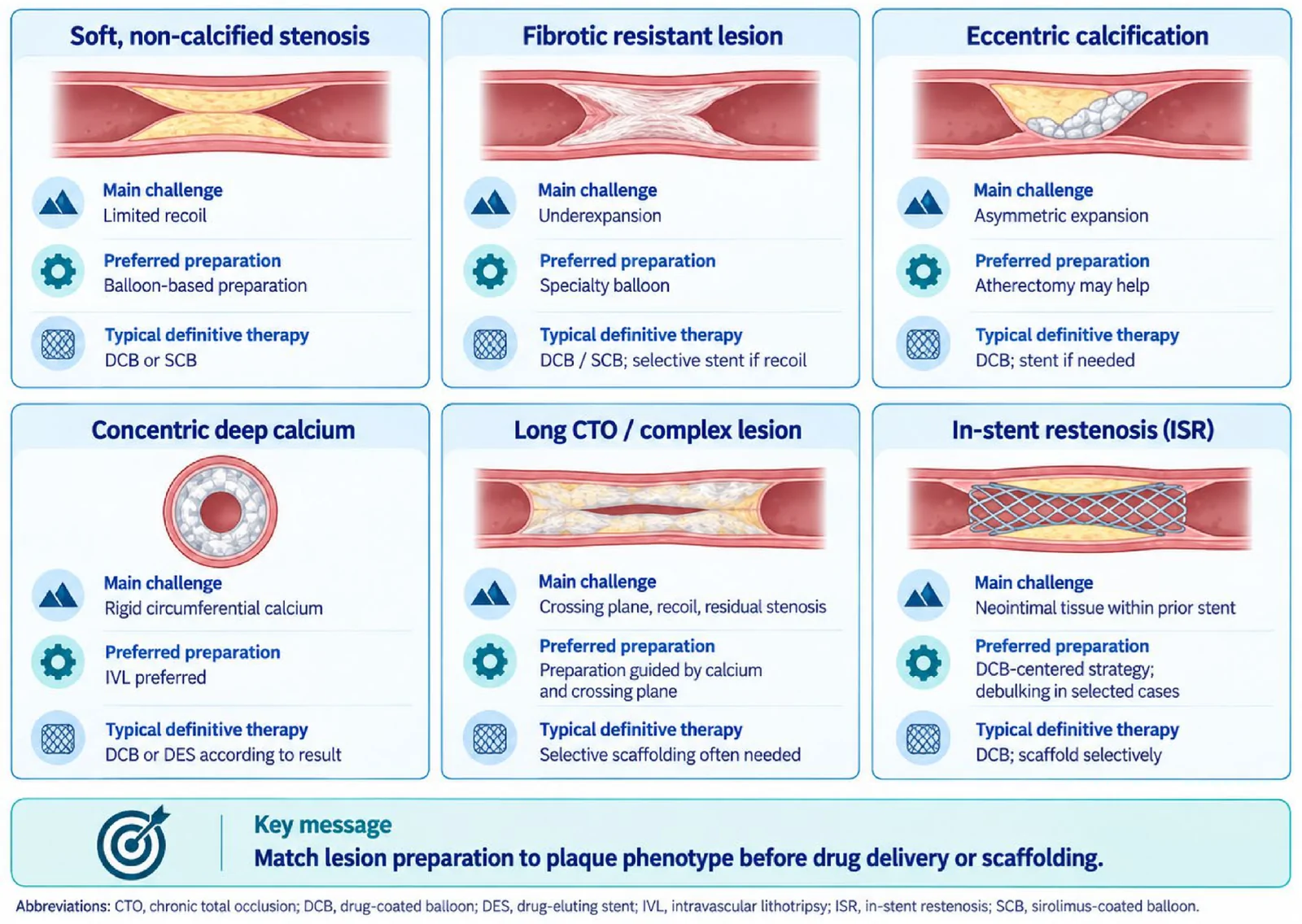
Plaque phenotype and lesion preparation strategies. Representative lesion phenotypes are aligned with the main mechanical challenge, the preferred preparation approach, and the most typical definitive therapy. The figure is intended as a practical framework rather than a rigid rule set: atherectomy may be useful in selected eccentric, calcified lesions, intravascular lithotripsy is particularly attractive for deep concentric calcium, and more complex lesions or in-stent restenosis often require selective scaffolding depending on the acute result. Abbreviations: CTO—chronic total occlusion; DCB—drug-coated balloon; DES—drug-eluting stent; ISR—in-stent restenosis; IVL—intravascular lithotripsy; SCB—sirolimus-coated balloon.

This lesion-oriented sequence—characterize the plaque, identify the dominant mechanical limitation, prepare selectively, reassess the acute result, and then choose antiproliferative therapy with or without scaffolding—provides the most coherent framework for contemporary femoropopliteal intervention. It also highlights the principal evidence gap: many technologies have been studied individually, but high-quality randomized comparisons of complete treatment strategies in clearly defined lesion phenotypes remain limited [1,8,9,10,11,12,13,14,15]. Representative plaque phenotypes and matched preparation strategies are summarized in Figure 2.

## 5. Clinical Integration: From Lesion Phenotype to Definitive Strategy

The practical value of a contemporary femoropopliteal strategy lies less in identifying a single superior device than in matching the dominant lesion problem to the least complex effective treatment. The sequence should begin with characterization of lesion length, occlusion, calcium distribution, vessel diameter, landing zones, and prior implants, followed by an explicit assessment of whether the lesion is likely to respond to balloon-based preparation alone. In straightforward, non-calcified stenoses, controlled balloon preparation followed by antiproliferative therapy remains a rational default. Randomized DCB trials established clear superiority over plain angioplasty for patency and repeat intervention, while more recent syntheses support the durability of drug-based therapy without a convincing contemporary mortality penalty [4,5,6,19,20,38]. Key randomized and pivotal studies supporting these contemporary strategies are summarized in Table 2.

As lesion complexity increases, the importance of preparation also increases. Long lesions and CTOs frequently combine diffuse plaque burden, variable vessel diameter, subintimal crossing, and greater risk of recoil or dissection. In this setting, the procedural objective should be to create a uniform lumen before deciding whether a leave-nothing-behind strategy is realistic. The available evidence does not justify routine atherectomy for every long or occlusive lesion, but it does support selective use when plaque burden or lesion compliance is the dominant mechanical obstacle. Randomized and pooled data consistently suggest that atherectomy can reduce severe dissection and bailout stenting, whereas improvement in long-term patency or clinically driven target-lesion revascularization is less consistent [9,10,11,12,39]. This distinction is important: a better acute angiographic result is clinically valuable, but it should not automatically be interpreted as proof of long-term superiority.

Heavily calcified disease deserves a separate pathway. Deep and circumferential calcium can prevent adequate balloon expansion and may reduce the effectiveness of subsequent drug delivery. In these lesions, intravascular lithotripsy is particularly attractive because it modifies calcium while preserving the vessel architecture. Disrupt PAD III demonstrated higher procedural success and markedly lower rates of flow-limiting dissection and provisional stenting with IVL than with conventional angioplasty in moderately to severely calcified femoropopliteal lesions [15]. Contemporary meta-analyses support this procedural advantage, although definitive evidence for superiority in long-term patency remains less mature [13,14]. When eccentric superficial calcium or bulky plaque predominates, debulking techniques may be more logical than IVL, emphasizing again that calcium morphology, not merely calcium presence, should guide the choice of preparation.

The decision between DCB and DES should likewise be framed by lesion behavior after preparation. If an adequate lumen is achieved without major recoil or flow-limiting dissection, DCB therapy preserves future options and avoids a permanent implant. Conversely, persistent recoil, major dissection, long segments with poor mechanical stability, or failed preparation favor scaffolding. Comparative meta-analyses suggest broadly similar medium-term effectiveness between primary DCB and DES in many native femoropopliteal lesions, with the balance determined by the need for a scaffold rather than by a universal efficacy hierarchy [28,29,40,41]. Drug-eluting stents remain particularly relevant in lesions in which sustained radial support and antiproliferative therapy are both desirable [42], while covered stents may have a role in selected long or recurrent disease. In mobile popliteal segments, however, permanent scaffolding should be considered cautiously because biomechanical deformation persists after implantation.

In-stent restenosis represents a special exception to the de novo treatment pathway. The presence of an existing scaffold changes both the biology and mechanics of the lesion. DCB therapy and covered-stent strategies outperform plain angioplasty in comparative evidence, while laser or other debulking approaches may be useful when diffuse tissue burden, occlusion, or underexpansion limits drug delivery [36,37]. The operator should therefore determine whether restenosis reflects predominantly neointimal proliferation, mechanical failure, or a combination of both before selecting therapy. Treating every ISR lesion with the same device is unlikely to address the underlying mechanism.

Finally, intravascular imaging can improve the quality of each of these decisions. IVUS identifies reference-vessel size, calcium distribution, plaque burden, wire position, residual disease, and device expansion more accurately than angiography alone. In the randomized trial by Allan et al., IVUS changed the procedural strategy in approximately four out of five cases and improved freedom from restenosis at 12 months [16]. These data support a broader concept: imaging should be used not as an isolated adjunct, but as a tool that increases the precision of lesion-oriented treatment, particularly in long, calcified, recurrent, or anatomically ambiguous disease. The resulting framework is deliberately simple: characterize the lesion, correct the dominant mechanical problem, deliver antiproliferative therapy when feasible, and implant a scaffold only when the vessel result requires it. A practical lesion-oriented treatment framework integrating lesion phenotype, preparation strategy, definitive therapy, and indications for scaffolding is provided in Table 3.

## 6. Limitations

This review has several limitations. First, it is a state-of-the-art narrative review rather than a formal systematic review; therefore, study selection was structured and evidence was prioritized hierarchically, but no PRISMA-based study-selection process or formal risk-of-bias assessment was performed. Second, the femoropopliteal literature is heterogeneous with respect to lesion length, calcification grading, inclusion of chronic total occlusions, clinical presentation, bailout-stenting criteria, definitions of primary patency, and follow-up duration. These differences limit direct comparison across technologies and are particularly relevant when interpreting network meta-analyses or indirect rankings. Third, much of the randomized evidence derives from patients with intermittent claudication and relatively selected anatomy, whereas patients with chronic limb-threatening ischemia, very long lesions, popliteal disease, severe calcification, and recurrent disease are underrepresented. Fourth, several newer technologies, particularly sirolimus-coated balloons and advanced plaque-modification strategies, have less mature long-term evidence than established paclitaxel-based therapies. Finally, technology evolves faster than comparative trial evidence, meaning that conclusions regarding individual devices should be interpreted within the generation of devices and techniques studied. The principal unresolved evidence gaps and priorities for future research are summarized in Table 4.

## 7. Conclusions

Contemporary endovascular treatment of femoropopliteal disease has moved beyond a device-first approach. The most coherent strategy is lesion-oriented: characterize the anatomy and plaque phenotype, identify the dominant mechanical limitation, perform selective vessel preparation, deliver antiproliferative therapy when an adequate lumen can be achieved, and reserve scaffolding for lesions with recoil, significant dissection, mechanical instability, or other features that make a leave-nothing-behind strategy unreliable. Atherectomy appears most useful as a selective preparation tool rather than a routine adjunct, whereas IVL has particularly strong procedural evidence in heavily calcified lesions. Paclitaxel-coated balloons remain an established therapy, while sirolimus-coated balloons now provide a credible alternative supported by randomized comparative data. Drug-eluting and other contemporary stents retain an essential role when durable mechanical support is required. Future trials should focus less on broad device-versus-device comparisons and more on well-defined lesion phenotypes, standardized morphology, intravascular imaging, long-term durability, and clinically meaningful outcomes. In practice, duplex ultrasound should establish baseline anatomy and follow-up; CTA or MRA can clarify complex or multilevel disease, and IVUS can refine sizing and assess calcium or an inadequate result. A short compliant stenosis may proceed from controlled balloon preparation to DCB, whereas deep circumferential calcium favors IVL before definitive therapy. For focal post-DCB dissection, consider localized repair when feasible; persistent recoil or extensive flow-limiting dissection warrants a suitable scaffold. In popliteal segments, minimize implant length while preserving an adequate final lumen. These choices should also reflect symptoms, limb threat, runoff, patient risk, and the availability of a suitable bypass conduit.

## Figures and Tables

**Table 2:** Key randomized and pivotal evidence in contemporary femoropopliteal endovascular therapy.

Study/Technology	Population	Comparator	Endpoint	Key Result	Clinical Take-Home
**IN.PACT SFA** [4]**|Paclitaxel DCB**	SFA/popliteal PAD	DCB vs. PTA	12-mo patency/CD-TLR	12-mo primary patency: 82.2% vs. 52.4% (*p* < 0.001); CD-TLR: 2.4% vs. 20.6% (*p* < 0.001) [4].	DCB became a definitive antiproliferative strategy rather than PTA alone.
**LEVANT 2** [5]**|Paclitaxel DCB**	476 symptomatic patients	Lutonix DCB vs. PTA	12-mo patency	65.2% vs. 52.6% (*p* = 0.02)	Confirms paclitaxel DCB superiority over plain angioplasty.
**ILLUMENATE** [6]**|Paclitaxel DCB**	Femoropopliteal disease	Stellarex DCB vs. PTA	12-mo patency/safety	Low-dose DCB superior to PTA	Supports efficacy across different paclitaxel DCB platforms.
**IMPERIAL** [7]**|DES**	Eluvia: polymer-coated paclitaxel-eluting DES; Zilver PTX: polymer-free paclitaxel-coated stent	Eluvia vs. Zilver PTX	12-mo safety/patency	12-mo primary patency: 86.8% vs. 81.5%; noninferiority for safety and efficacy [7].	DES is reasonable when antiproliferative therapy and durable scaffolding are both needed.
**DEFINITIVE AR** [39]**|Atherectomy + DCB**	Randomized pilot	Atherectomy + DCB vs. DCB	Acute/12-mo outcomes	Better acute preparation; not powered for durable superiority	Supports selective, not routine, atherectomy before DCB.
**Disrupt PAD III** [15]**|IVL**	306 calcified lesions	IVL vs. PTA before definitive therapy	Procedural success	65.8% vs. 50.4%; less dissection/stenting	Strongest randomized evidence for calcium-specific preparation.
**Allan et al.** [16]**|IVUS**	150 interventions	IVUS- vs. angiography-guided	12-mo restenosis	72.4% vs. 55.4%; strategy changed in 79%	IVUS is most useful when characterization or optimization can change treatment.
**SIRONA** [21]**|Sirolimus DCB**	482 patients; mean lesion 8.4 cm; CTO 33.2%	Sirolimus vs. paclitaxel DCB	12-mo patency	73.9% vs. 74.9%; noninferiority met	Supports sirolimus DCB as a contemporary alternative to paclitaxel.
**SIRONA 2-y** [22]**|Sirolimus DCB**	24-mo follow-up	Sirolimus vs. paclitaxel DCB	Patency/CD-TLR	Patency 64.6% vs. 67.2%; freedom from CD-TLR 91.2% vs. 88.1%	Comparable 2-y outcomes; longer-term durability remains less mature.

**Table 3:** Practical lesion-oriented endovascular strategy according to femoropopliteal lesion phenotype. Recommendations reflect cited evidence and expert interpretation; they are not validated treatment rules.

Lesion Scenario	Dominant Problem	Preferred Preparation	Definitive Strategy	Scaffold Trigger/Key Caveat
**Short, non-calcified stenosis**	Recoil or dissection after simple dilatation	Conventional balloon preparation	DCB if final mechanical result is satisfactory	Scaffold for persistent recoil, flow-limiting dissection or major residual stenosis. Best setting for leave-nothing-behind. Evidence: [4,5,6,32].
**Fibrotic/balloon-resistant lesion**	Persistent waist or underexpansion	Prolonged/high-pressure angioplasty; consider scoring/cutting balloon	DCB once adequate expansion is achieved	Escalate preparation before implanting a scaffold solely because the first balloon result is suboptimal. Evidence: [1,8,9].
**Long lesion/CTO**	Plaque burden, subintimal crossing, dissection/recoil and lower durability	Consider IVUS; plan crossing; selective plaque modification	DCB with selective scaffold, or DES/covered stent when support is anticipated	Plan bailout in advance. Long spiral dissection, recoil, inadequate lumen or flow limitation favor scaffolding [26,27,28]. Focal dissection repair may limit implant length after DCB [35].
**Severe eccentric/bulky calcium**	Asymmetric noncompliance and plaque burden	IVUS where available; selective directional/rotational atherectomy	DCB if adequate lumen/expansion are achieved	Residual stenosis/recoil or major dissection may require scaffold. Durable atherectomy superiority is unproven [9,10,11,12,39].
**Circumferential/deep calcium**	Failure of expansion due to deep calcium	IVUS where available; IVL	DCB or DES according to final mechanical result	Persistent recoil/underexpansion despite calcium modification favors scaffold. IVL has strongest RCT support [13,14,15]. Evidence: [15,31].
**Popliteal involvement**	Flexion, torsion, compression and elongation	Atraumatic preparation; selective intravascular imaging	DCB when result is satisfactory	Prefer the shortest effective repair. For focal flow-limiting post-DCB dissection, consider a nitinol tack where suitable (single-arm evidence [35]); longer dissection or recoil may require bailout bare-metal or drug-eluting stenting.
**Femoropopliteal ISR**	Hyperplasia, neoatherosclerosis, underexpansion or stent failure	Define failure mechanism; consider IVUS; selective laser/debulking	DCB or covered stent; adjunctive debulking selectively	Mechanical stent failure or recurrent diffuse ISR may require relining. POBA alone has high recurrence [36,37].
**CLTI/high-risk limb**	Need for durable perfusion; multilevel disease; limited trial generalizability	Assess inflow/outflow and limb strategy; use imaging selectively	Choose DCB/DES/scaffold according to mechanics and revascularization goal	Lower threshold for durable support when bailout failure would threaten limb perfusion; prioritize limb-based outcomes. Evidence is extrapolated because CLTI was underrepresented in pivotal trials [2,3].

**Table 4:** Current evidence gaps and future priorities in femoropopliteal endovascular therapy. Examples refer to the studies discussed in the text; proposed priorities are the authors’ interpretation.

Domain	Current Limitation	Why It Matters	Research Priority
**Atherectomy + DCB**	Procedural benefit is more consistent than durable clinical superiority	Routine use may add cost/embolic risk without proven long-term benefit in all lesions	Morphology-stratified head-to-head RCTs.
**IVL**	Strong randomized procedural benefit; limited incremental long-term patency data	Better acute expansion may not necessarily translate into durable clinical benefit	Long-term comparative and cost-effectiveness studies.
**Sirolimus DCB**	Noninferiority vs. paclitaxel through 2 years; less mature long-term evidence	Future choice will depend on durability, not only early efficacy	Long-term follow-up and complex-lesion trials.
**IVUS-guided therapy**	Treatment modification/restenosis benefit supported; routine-use economics uncertain	Value may depend strongly on lesion complexity	Pragmatic trials defining high-yield indications and cost-effectiveness.
**Long lesions/CTO**	Durability remains lower; direct strategy comparisons are sparse	Uncertainty persists between bailout-only and planned scaffolding	Direct DCB vs. DES/covered-stent strategy trials.
**Popliteal disease**	Few dedicated randomized studies	Biomechanics may alter the risk–benefit balance of permanent implants	Popliteal-specific prospective trials. Consider focal dissection repair versus conventional bailout stenting in prospective comparisons [35].
**ISR**	Different failure mechanisms are commonly pooled together	Hyperplasia, underexpansion, fracture and occlusion may require different therapies	Imaging-defined, mechanism-specific ISR trials.
**CLTI**	Underrepresented in pivotal device trials	Patency alone misses wound healing, limb salvage and survival trade-offs	CLTI-specific trials with limb and patient-centered outcomes.

## Data Availability

No new data were created or analyzed in this study. Data sharing is not applicable to this article. The publications supporting this review are available from the corresponding publishers and bibliographic databases.
